# 788. *Enterobacter cloacae* Infection Characteristics and Outcomes in Military Personnel who Sustained Trauma in Iraq and Afghanistan

**DOI:** 10.1093/ofid/ofab466.985

**Published:** 2021-12-04

**Authors:** William N Bennett, Joseph Yabes, Katrin Mende, Miriam Beckius, Azizur Rahman, David Tribble

**Affiliations:** 1 SAUSHEC, San Antonio, Texas; 2 Brooke Army Medical Center, San Antonio, Texas; 3 Infectious Disease Clinical Research Program, Bethesda, MD, The Henry M. Jackson Foundation, Bethesda, MD, and Brooke Army Medical Center, Fort Sam Houston, TX, San Antonio, TX; 4 Brooke Army Medical Center, JBSA Fort Sam Houston, TX, USA, San Antonio, TX; 5 Infectious Diseases Clinical Research Program, Uniformed Services University of the Health Sciences, Bethesda, Maryland; 6 Uniformed Services University, Bethesda, MD

## Abstract

**Background:**

*Enterobacter cloacae* is a Gram-negative rod with chromosomally-induced Amp-C β-lactamase with multidrug-resistant potential. Joint Trauma System guidelines for treating combat wounds include prophylaxis with cefazolin and ertapenem, potent inducers of Amp-C. We evaluated clinical characteristics, antibiotic utilization, and outcomes associated with battlefield-related *E. cloacae* infections.

**Methods:**

All initial solitary (those with single isolates) and serial *E. cloacae* isolates (≥24 hours from initial isolate from any site) were collected from the Trauma Infectious Disease Outcomes Study (6/2009-12/2014). Inclusion required *E. cloacae* isolation from a clinical infection. Amp-C-inducing β-lactams were classified based on induction potential and lability to the Amp-C β-lactamase as Amp-C induction levels.

**Results:**

Of 653 *E. cloacae* isolates, 253 met inclusion criteria – 64 patients had only initial isolates, 54 patients had serial isolates. Patients were largely male (99%), median age 23 years (IQR 21-27), with injury severity score of 34 (IQR 24-45). Initial isolates were wound (70%), respiratory (22%), blood (7%), urine (1%), and other (1%). Patients commonly had blast injuries (89%), required ICU admission (95%), and had a median hospital stay of 57 days (IQR 39-82). Patients with serial isolates showed a trend towards earlier clinical infection (5 vs 8 days, P = 0.07). They were also less likely to receive carbapenems prior to *E. cloacae* isolation compared to those with only initial isolates (4% vs 38%) and more likely to receive 1^st^ generation cephalosporins (79% vs 58%, P = 0.01). The serial isolate group received more days of 1^st^ generation cephalosporins (median 6 days vs 2.5 days, P = 0.01). Cumulative antimicrobial therapy trended towards significance and was greater with the serial isolates (median 100 days vs 74 days, P = 0.08). There was no difference in number of surgical interventions between those with and without serial isolates (P = 0.54). Overall, 6 patients died.

**Conclusion:**

*E. cloacae* infections after battlefield trauma were frequently encountered and associated with exposure to 1^st^ generation cephalosporins. Serial infections did not correlate to worse patient outcomes but displayed a trend towards an overall greater duration of antibiotic use.

**Disclosures:**

**William N. Bennett, V, MD**, **Abbvie** (Shareholder)**Amgen** (Shareholder)**Nabriva** (Shareholder)

